# Immune modulation after traumatic brain injury

**DOI:** 10.3389/fmed.2022.995044

**Published:** 2022-12-01

**Authors:** Marwan Bouras, Karim Asehnoune, Antoine Roquilly

**Affiliations:** ^1^Nantes Université, CHU Nantes, INSERM, Center for Research in Transplantation and Translational Immunology, UMR 1064, Nantes, France; ^2^CHU Nantes, INSERM, Nantes Université, Anesthesie Reanimation, CIC 1413, Nantes, France

**Keywords:** traumatic brain injury, innate immunity, inflammation, immunosuppression, immunomodulatory therapy

## Abstract

Traumatic brain injury (TBI) induces instant activation of innate immunity in brain tissue, followed by a systematization of the inflammatory response. The subsequent response, evolved to limit an overwhelming systemic inflammatory response and to induce healing, involves the autonomic nervous system, hormonal systems, and the regulation of immune cells. This physiological response induces an immunosuppression and tolerance state that promotes to the occurrence of secondary infections. This review describes the immunological consequences of TBI and highlights potential novel therapeutic approaches using immune modulation to restore homeostasis between the nervous system and innate immunity.

## Introduction

Traumatic brain injury (TBI) is a significant cause of morbidity and mortality, mainly in young patients (1). In addition to the high mortality in the immediate aftermath, the severity of these traumas is related to the significant morbidity and mortality in intensive care units (ICU) (2). While most of the morbidity and mortality factors are direct consequences of the trauma (initial severity of injury, secondary bleeding), approximately one-third are represented by hospital-acquired infectious complications (3). Around 50% of patients with severe TBI mechanically ventilated in ICU will develop ventilatory-associated pneumonia (4). Patients with severe TBI complicated by hospital-acquired infection develop more secondary systemic brain aggressions (sepsis, hypoxia, hypercapnia) that lead to intracranial hypertension and further brain damage, prolonged mechanical ventilation, and ICU length of stay (5) and is an independent risk factor for unfavorable neurological outcome (6).

The susceptibility of patients with TBI to nosocomial infections is correlated with the development of a state of immunosuppression that sets in in the direct aftermath of the trauma (7) some characteristics of which are close to the immunosuppression found in septic shock (8). Following a TBI, producing proinflammatory cytokines is a necessary physiological phenomenon that promotes the healing of contused tissues and the defense against developing secondary infections. However, in some patients, the development of an exacerbated systemic inflammatory response syndrome (SIRS) can induce multiple organ failure syndromes with a very high mortality rate (9). To prevent the occurrence of an exacerbated proinflammatory response, the central nervous system (CNS), in association with innate immunity, initiates a systemic anti-inflammatory response (compensatory anti-inflammatory response [CARS]). This response aims to restore homeostasis but increases the risk of post-traumatic infectious complications (10). Studying the immune modulation after TBI, is necessary to propose relevant therapeutic approaches to reduce the morbidity and mortality of these patients.

## Intracerebral immune response

After TBI, meningeal contusion, axonal shearing, or cerebrovascular injury alter the functions of glial cells for days (11). The release and extracellular accumulation of intracellular components (such as ATP, HSPs, and HMGB1…), which are recognized as Damage-Associated Molecular Patterns (DAMP), activate innate immune receptors such as Toll-Like Receptors (TLR) expressed on glial cells, macrophages, dendritic and endothelial cells, and astrocytes (12). When exposed to DAMP, microglia cells are rapidly activated to clear debris, reconstitute the defective blood-brain barrier (BBB), and produce nutritional factors for the brain cells (13). At the same time, microglia produces proinflammatory cytokines such as IL-1β and IL-6, which recruit neutrophils and blood monocytes-macrophages to the injured area (14). Neutrophils cross the BBB within minutes after TBI and DAMPs such as HMGB1 from necrotic neurons increased leukocytes activation, IL-6 secretion via TLR4 pathway and induce brain edema (15). Activated neutrophils subsequently produce NETs, web-like chromatin structures, which prime other immune cells to induce sterile inflammation (16). In humans TBI, NETs formation coincide with cerebral hypoperfusion and tissue hypoxia (17). The release of HMGB1 from necrotic neurons may initiate a TLR4-dependent NETs formation and promote neuroinflammation by IL1- β and IL-6 secretion from peripheral blood mononuclear cells (PBMC). After exposure to IL-1β, astrocytes rapidly generate immune signals, resulting in more neutrophils recruitment and systemic cytokine release (18). Immigrating neutrophils also help to remove debris such as myelin fragments and exacerbate inflammation and neuronal loss (19). This activation induces the production of proinflammatory cytokines and stimulates antigen presentation by antigen-presenting cells (APCs) to effector cells (T and Natural Killer (NK) cells) (20).

At the same time, trauma-activated platelets and the subsequent coagulation cascade release pro-inflammatory mediators interacting with the immune system (21) and generating a self-perpetuating cycle of local inflammation. Activated platelets form aggregates with leukocytes causing endothelial cell damage (22). Platelets and neutrophils are also significant sources of microvesicles and exosomes, which may contain various DAMPs (23) thereby propagating the inflammation to the systemic compartment (24).

Other pathophysiological consequences include blood-brain barrier disruption, cellular swelling and vasogenic edema. HMGB-1 released by necrotic neurons induces the production of IL-6 by microglia resulting in aquaporin water channel expression in astrocytes and cytotoxic swelling (15). In TBI, permeabilization of the BBB also leads to contact of CNS epitopes (such as myelin basic protein) with innate immune cells. Recognition of these antigens as DAMPs could lead to the maintenance of the local inflammatory state (25). DAMPs activate the complement cascade, leading to the rapid generation of C3a and C5a (26). Activation of complement and inflammatory cells triggers the production and release of inflammatory mediators such as interleukins, generating the systemic response seen in SIRS.

**FIGURE 1:**
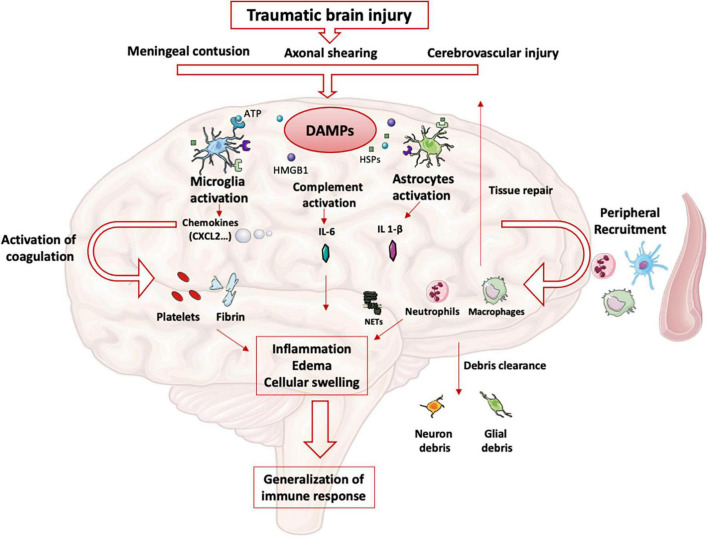
Innate immune response in brain: After TBI, parenchymal, vascular, and blood-brain barrier damage results in the production of DAMPs. These DAMPs activate resident brain cells (microglia, astrocytes) that initiate the intracerebral inflammatory response. The production of cytokines and chemokines leads to the recruitment of blood leukocytes and the activation of complement and coagulation pathways. All these phenomena maintain the state of intracerebral inflammation which causes edema and induces a systematization of the immune response.

The important release of neurotransmitters (glutamate, adenosine) in the extracellular space induces changes in the anti- and pro-inflammatory functions of glial cells and modulate neuroinflammation via numerous signaling pathways (i.e., the adenosine-A2AR system in mice (27). This balanced inflammatory response is intended to enable DAMPs clearance and induce tissue repair mechanisms by reprogramming brain macrophages from pro-inflammatory to anti-inflammatory functions (28). The CNS also induces a systemic anti-inflammatory response to avoid an exacerbated pro-inflammatory response, likely to cause multiple organ failure syndrome. This systemic immune response mainly involves innate immune cells and neurohormonal mechanisms (Figure 1). However, protracted anti-inflammatory response is associated with secondary infections.

## Systemic immune response

### Neurohormonal mechanisms

#### The sympathetic nervous system

In healthy conditions, the sympathetic nervous system plays an essential interface between the neural and immune systems (29). Post-ganglionic sympathetic nerves, which pass through the paraspinal and pre-spinal ganglia, release norepinephrine into primary and secondary lymphoid organs.

The sympathetic nervous system activation, which belongs to the systemic inflammatory response observed after TBI, results in the secretion of catecholamines into the periphery (30) in a dose-dependent manner according to the trauma severity. Blood catecholamine levels were significantly increased in peripheral circulatory system at the earlier stage of TBI and is correlated with adverse outcomes (31).

The sympathetic nervous system afferences are the primary and secondary lymphoid organs, including the thymus, bone marrow, spleen, lymph nodes, and mucosa-associated lymphoid tissues. The extensive sympathetic innervation of immune organs and the expression of adrenergic receptors on numerous leukocyte families suggest that immune function can be finely tuned by the level of sympathetic activity (32). Activation of the sympathetic system results in the release of catecholamines from sympathetic nerve endings and selectively inhibits interferon (IFN)-γ and IL-2 production by human Th1 T cells (33). Several studies have shown that norepinephrine and epinephrine secretions decrease the production of TNFα, IL-1β, and IL-12 by lipopolysaccharide (LPS)-stimulated mice monocytes (34) and that the activity of NK cells, including cytotoxic activity and production of effector cytokines, was inhibited by such catecholamines (35). In severe trauma patients, sympathetic nerve blockade, notably via the administration of beta-blockers, reduces the blood levels of proinflammatory cytokines such as TNFα, IL-1b, and IL-6 after brain injury (36, 37). Elevated levels of the anti-inflammatory cytokine IL-10 have been documented in patients undergoing a “sympathetic storm” in the aftermath of TBI. Blocking β-adrenergic receptors with a β -blocker such as propranolol provides a dose-dependent inhibition of this exacerbated IL-10 release in rats (38). In human, propranolol decreases in-hospital mortality and improves long-term functional outcome in isolated severe TBI (39) confirmed by a randomized controlled trial (40).

**FIGURE 2:**
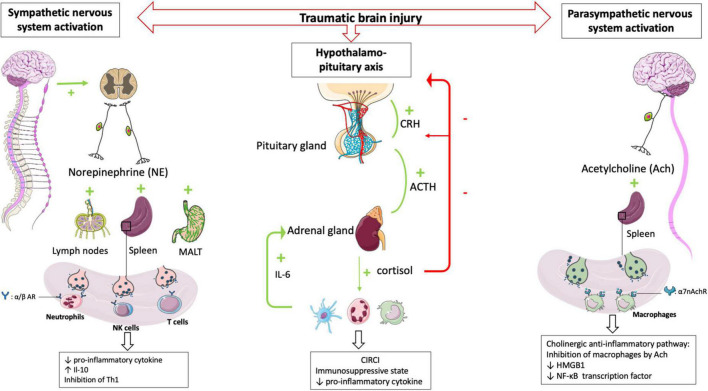
HPA axis and autonomic nervous system (i.e., sympathetic nervous system and parasympathetic nervous system) plays central role in regulating peripheral immune cells after TBI. After a TBI, the sympathetic and parasympathetic nervous systems stimulate the secondary lymphoid organs via their neurotransmitters (norepinephrine and acetycholine respectively) leading to a state of immunodepression due to the loss of inflammatory functions of the leukocytes. Post-traumatic activation of HPA axis leads to CIRCI and deepens the immunosuppressive state. α/β *AR*, α/β adrenergic receptor; α*7nAchR*, α*7* subunit of the acetyl choline receptor.

In a mouse model of focal cerebral ischemia, blockade of the sympathetic nervous system restores IFN-γ secretory capacity and decreases systemic bacterial infections (41). These data suggest that catecholamine secretion by the sympathetic system triggered by TBI contributes to the severe immunosuppression observed following TBI (Figure 2).

#### The parasympathetic nervous system

The parasympathetic nervous system is mainly composed of the vagus nerve, which innervates the liver, lungs, spleen, kidneys, and gut. There is also evidence for vagal innervation of lymphoid organs (42), the inhibitory role of which has been referred to as “the cholinergic anti-inflammatory pathway” (43). Thus Borovikova et al. demonstrated that in response to acetylcholine, activated macrophages decrease their production of proinflammatory cytokines such as TNFα, IL-1β, and IL-18, but not their secretion of anti-inflammatory cytokines such as IL-10 (44). The same authors (45) demonstrated that electrical stimulation of the peripheral vagus nerve decreased serum and liver levels of proinflammatory cytokine TNFα. Activating the parasympathetic nervous system in animal models decreases ischemia-reperfusion syndrome (46) and inflammatory state after hemorrhagic shock (47). Studies in deficient mice have provided additional evidence demonstrating a role for the α7 subunit of the ACh receptor (α7nAchR) on macrophages in the cholinergic anti-inflammatory pathway (48). The binding of ACh to this macrophage receptor results in activation of Jak2, which phosphorylates the DNA-binding transcription factor STAT3 (49), decreases the nuclear translocation of the transcription factor NF-κB and reduces the transcription of the DNA-binding protein HMGB1 (50), finally favoring anti-inflammatory response. These findings support that the activated efferent parasympathetic nervous system modulates systemic the immune responses as well as it regulates heart rate and other vital functions (51) (Figure 2).

#### The hypothalamo-pituitary axis and the critical illness-related corticosteroid insufficiency

The hypothalamic-pituitary-adrenal axis (HPA axis) is a complex neuroendocrine system controlling stress responses and regulating many vital functions. In response to stress the hypothalamic release of corticotropin-releasing hormone (CRH) is increased which leads to the secretion of the pituitary adrenéno-coticotropic-hormone (ACTH). In turn, the adrenal cortex synthesizes and releases cortisol into the circulation (52). Cortisol acts on all immune cells and elicits immunosuppressive and anti-inflammatory functions through genomic and non-genomic mechanisms (53). Hence, the combined activities of the autonomic nervous system and HPA axis play an essential role in regulating the immune system (54). In TBI patients the direct stimulation of the pituitary secretion of ACTH influences the number of β-adrenergic receptors on immune cells (55). Activation of β2-adrenergic receptors potentiates the expression of the glucocorticoid receptor gene through the activation of intracytoplasmic downstream pathways (56). After that, the HPA axis and sympathetic nervous system increase the release of cortisol and norepinephrine, contributing to the downregulation of immune cell activity (57) including the inhibition of innate and adaptive immunity and induction of leucocytes apoptosis.

Post-traumatic activation of the HPA axis usually results in the simultaneous release of dehydroepiandrosterone (DHEA). However, in the immediate aftermath of TBI, cortisol levels remain highly predominant over DHEA levels (58). The increase in the cortisol/DHEA ratio leads to a state of immunodepression, notably by inhibition of neutrophil function, and is associated with an increase in nosocomial infections (59). DHEA supplementation effectively reduces sepsis-related mortality in mice (60) and some preliminary data obtained in humans has strengthened the rationale to test this molecule in large clinical trials (61).

Rapidly after a TBI, there is a maladaptive secretion and abnormal cortisol response, so-called critical illness-related corticosteroid insufficiency (CIRCI). CIRCI is the impairment of the HPA axis secondary to inflammation which cannot respond adequately to secondary stimulations (62). Corticosteroid insufficiency occurs in 50-75% of patients with severe TBI and is associated with a poor prognosis (63).

Three major pathophysiological events account for CIRCI: anatomical disconnection of the HPA axis, alteration of the cortisol metabolism with decreased cortisol degradation (64), and tissue resistance to corticosteroids (65). The occurrence of CIRCI after TBI is associated with an anti-inflammatory state inappropriate to the severity of the disease (66), longer durations of vasopressor support, and poorer long-term neurological outcomes (67). After severe TBI, alteration of the HPA axis is a consequence of the post-traumatic cytokine storm (68) significantly influenced by IL-6 secretion. For example, the administration of human IL-6 increases plasma cortisol concentrations in mice (69). During activation of the immune system, such as during TBI, it has been shown that IL-6 is a potent ACTH-independent stimulator of the HPA axis (70). In children with TBI, serum IL-6 levels are elevated and correlated with the severity of the TBI, and increased IL-6 levels are associated with a significant systemic inflammatory response that can lead to the development of ARDS (71). Structural damage to the pituitary glands can also cause CIRCI in TBI patients (72) inducing the loss of blood-brain barrier permeability and apoptosis of hypothalamic neurons. After TBI, corticosteroid insufficiency aggravates the state of immunosuppression and increases the risk of the secondary respiratory infections (73). However, the principle of CIRCI and the resulting modulation of the HPA axis has been the subject of many therapeutic trials, many of which have failed to show efficacy for patients (74) including the largest RCT to investigate the impact on outcome of glucocorticoid treatment of patients with septic shock (75).

### Immune cells and traumatic brain injury

**FIGURE 3:**
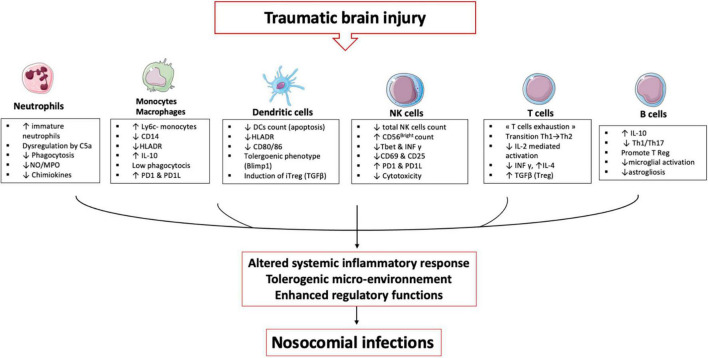
TBI induces alterations in immune cells: These immune alterations concern myeloid and lymphoid cells, starting in the bone marrow and lymphoid organs and then in the circulating cells. It is an alteration of the first line of defense (neutrophils) and an inability of the monocyte & macrophages to maintain the inflammatory response. Dendritic cells lose their ability to present antigen and secrete pro-inflammatory cytokines. All these cells develop a tolerogenic phenotype unable to initiate the lymphocyte response, which leads to an expansion of regulatory T cells. All these mechanisms induce a phenomenon called post-traumatic immunodepression and favors the occurrence of nosocomial infections.

TBI induces alterations in immune cells and each plays an important role in establishing an immunosuppression and tolerance state (Figure 3).

#### Neutrophils

An increased neutrophil count is observed up to 48 h after TBI (76) notably through a delayed apoptosis mechanism (77) as well as endogenous release of cortisol and catecholamines promoting neutrophil demargination and their exit from the bone marrow (78). TBI is therefore characterized by circulating neutrophil populations at different stages of maturation. In the homeostatic state, a single population of mature neutrophils (CD16^bright^) circulates in the peripheral blood and trauma is accompanied by a significant release of immature neutrophils (CD16^dim/^) into the circulation (79). At the same time, there is a significant release of myeloid-derived suppressor cells (MDSCs) through a CXCL2-dependent mechanism, which deepens the immunosuppressive state (80). In the aftermath of TBI, neutrophils also show phenotypic changes affecting their effector functions. There is a reduced expression of the IL-8 receptors CXCR1 and CXCR2 in blunt chest injured patients (81) as well as CD11b (a component of the β2 integrin receptor MAC-1), which is involved in neutrophil membrane adhesion (82). When stimulated *in vitro* with fMLP, (a neutrophil activator), neutrophils from TBI patients do not upregulate the CD11b and FcγRII receptors (83) explaining the hypo responsiveness of circulating neutrophils to bacterial stimulation. In the hours and days following severe TBI, neutrophils show an increase in ROS production in the resting state (84) and response to stimulation (77). Loss of regulatory feedback on immune function following direct injury to the CNS appears to be the primary cause. After that, neutrophils exhibit reduced ROS production in the days following TBI (85) associated with impaired phagocytic abilities. Thus, after TBI, the capacity of circulating neutrophils to phagocytose *Escherichia coli* is significantly reduced for up to several weeks after the trauma. The reduction in phagocytosis of extracellular bacteria is more significant after TBI than after severe trauma without CNS injury, suggesting a compensatory anti-inflammatory mechanisms exacerbated to protect brain tissue (84). Complement activation is an immediate response to trauma and is correlate with the severity of the trauma and the occurrence of infections (86). The C5a fraction of complement, a critical mediator allowing neutrophils to phagocytose bacteria (87), may be one cause of the neutrophil anergy and critical illness induced organ dysfunction (88). Indeed, complement activation is a hallmark of the inflammatory response to TBI (89) and in a mouse model of TBI, C5a influences the functional behavior of circulating neutrophils (90). Critically ill patients exhibit significant circulating neutrophil dysfunctions, which is mediated by activated complement like C5a which is known to reduce chemotaxis, respiratory burst, and phagocytosis (91, 92). Trauma also induces an immediate alteration of leukocyte receptors to C5a (CD88) (93) indicating a likely multi-factorial mechanism inducing neutrophil anergy as found in human sepsis (94). Studies of neutrophils from ICU patients and healthy volunteers demonstrate that C5a induces a prolonged defect in phagocytosis of relevant pathogens (*S. aureus* and *E. coli*) persisting for several hours and also induces a defective phagosomal maturation thus decreasing the anti-bacterial capacities of neutrophils (95). Another neutrophil-derived mediator of the post-trauma inflammatory response is NETs (96). As seen above, neutrophils in the brain parenchyma produce NETs in the immediate aftermath of trauma (97, 98). NETs are essential in the capture and the antibacterial defense but in the aseptic inflammation caused by TBI, the presence of histones and extracellular peptides may have detrimental effects. *In vitro*, NETs induce lung epithelial and endothelial cell death in mice (99), whereas *in vivo*, NETs appear to promote deep vein thrombosis by intravascular inflammation, a secondary complication with high morbidity in TBI patients (100).

Neutrophils are therefore pivotal cells and some authors have shown that dysfunctions affecting these cells are correlated with organ failure and secondary infections in trauma patients and may represent a promising avenue for the development of immune dysfunction biomarkers (101).

#### Monocytes/macrophages

In TBI patients, monocytes/macrophages undergo many modifications and it is worth noting the difference between microglia, monocyte-derived macrophages infiltrating the cerebral tissue, and circulating macrophages.

Microglia represent the resident macrophages of the CNS and, as seen previously, are the first immune cells to be affected by TBI. Microglia have a great potential to adapt to inflammatory conditions and can develop or annihilate some functions (102). After their activation, the microglia cells migrate to the site of injury, undergo morphological changes (to form larger cell bodies with ramified cellular structures) and changes in pro and anti-inflammatory functions (103). For a long time, authors have described, based on *in vitro* studies (104), two states of polarization of the microglia after their activation. The M1 phenotype would have a pro-inflammatory function and secretion of chemokines (105) while the M2 phenotype would have an anti-inflammatory and neuroprotective role (106). However, this dichotomy does not represent the reality *in vivo* which is more complex (107). M1 phenotype can secrete anti-inflammatory cytokine such IL-10, and the M2 phenotype can secret pro-inflammatory cytokines. Recent publications (108) including transcriptomic analyses in animal models (109) show that several microglia phenotypes coexist and that TBI induces multiple responses in microglia including electrophysiological changes, proliferation, migration, release of cytokines/chemokines, and phagocytosis (110). This results in the release of many factors into the injured tissue, such as macrophage colony-stimulating factor (M-CSF), brain-derived neurotrophic factor (BDNF) or neurotrophin 3 (NT-3). Microglia also plays a role in phagocytosis of dead cells. via the P2Y6 receptor that detects UDP released from dead cells (111). After activation, microglia produce inflammatory mediators such as IL-1b, IL-6, IL-12, NO or ROS (11). The production of these mediators may promote the inflammatory response by increasing BBB permeability and facilitating the recruitment of peripheral immune cells (112). This recruitment is also mediated by the microglia secretion of extracellular vesicles that sustain neuroinflammation and induce cell communication after TBI (113) resulting in the migration of peripheric blood monocytes/macrophages.

The arrival of monocytes and their subsequent transformation into macrophages in the injured brain occurs within 24 hours after TBI (114). One mechanism of monocyte recruitment after TBI relies on the production of CCL2 in the CSF by the choroid plexus epithelium (115). Recent transcriptomic studies have differentiated infiltrating monocytes-derived macrophages from resident microglia cells (116) and animals models have hypothesized that in the days following TBI, the infiltrating macrophages induce damages by exacerbated inflammation while the glial cells may have a primarily neuroprotective effect. Hence, in a rat model of TBI the production of inflammatory mediators (IL-1β, and CD68) were higher in macrophages, whereas the TGF-β1 was higher in microglia (117). Macrophages may be considered the aggravating cell type, whereas activated microglia may play a favorable role during the acute phase of TBI.

The effects of TBI on the circulating monocyte numbers differed between animal and human studies. In contrast to the murine studies, where significant reductions in monocyte numbers were observed in the first few hours after TBI (118), the human studies showed a substantial increase in the absolute number of circulating monocytes (84). In mice, classical Ly6C + monocytes secrete proinflammatory cytokines and differentiate into proinflammatory macrophages after getting into the target tissue. In contrast, non-classical Ly6C- monocytes secrete the anti-inflammatory cytokine IL-10 and develop anti-inflammatory functions (119). In a mouse model of TBI, Schwulst et al. found a significantly higher number of anti-inflammatory Ly6C- monocytes in the peripheral circulation. Consistent with this observation, intracellular expression of IL-10 was detected in monocytes isolated from TBI patients immediately after injury (120), suggesting that TBI causes tolerogenic polarization of circulating macrophages, leading to an anti-inflammatory response. In the lungs, after the resolution of acute inflammation caused by TBI, alveolar macrophages present with a low phagocytic capacity for several weeks. With murine models our team to demonstrate that these paralyzed alveolar macrophages are developed from resident macrophages that undergo tolerogenic epigenetic reprogramming *in situ* (121). This adaptation was not induced by a direct encounter with pathogens or DAMP but by locally established secondary immunosuppressive signals after the resolution of the primary inflammation. These experimental data on animal models have been confirmed on samples of ICU patients. Hence, circulating monocytes of patients with severe trauma expressed altered levels of regulators of phagocytosis CD14, CD16 and Signal regulatory protein α (SIRPα) for months after the insult. SIRPα plays a critical role in establishing the microenvironment that induces tolerogenic formation in critically ill patients with significant systemic inflammation like TBI patients, circulating monocytes exhibiting alterations consistent with this immunosuppressive reprogramming six months after resolution of inflammation. Despite discrepancies between mice and humans data, several of these monocytic alterations have been validated in patients with TBI, including low phagocytic capacity (121), low capacity to activate natural killer cells via the production of IL-12 (122), and more recently a transcriptomic signatures has been identified in monocytes and associated with Herpes Simplex Virus lung reactivation and unfavorable neurological outcomes (123). As summary, the monocytes-macrophages, which continuously adapt their functions to spatiotemporal modifications of their microenvironment (124), appear central to the immune adaptation to TBI.

#### Dendritic cells

Dendritic cells (DC) count and maturation status are affected in ICU patients (125). A reduction in the number of circulating DC has been observed in brain-injured patients (126) as well as in the pool of resident DC in lymphoid organs. In addition to this decrease, the systemic circulation of DAMP after TBI activate immature DC, leading to a reduction in their ability to present antigens encountered later, impairing the power of the immune system to respond to secondary insults (127). The induction of “tolerogenic” DC, characterized by Blimp-1 expression, low antigen presentation, and IL-12 production capacities, is a significant mediator of post-traumatic susceptibility to infections. These tolerogenic DCs induce an immunosuppressive microenvironment, notably conventional type 1 DCs produce anti-inflammatory cytokines and induce the peripheric conversion of CD4 effector T cells in regulatory FoxP3 T cells (iTregs) (128). In the aftermath of TBI, Th2-inducing DCs may counteract Th1-type inflammation, thereby regulating inflammation. The immunogenic or tolerogenic characteristics of DCs may be directly related to the developing concept of “innate memory,” where “entrained” DC might exhibit increased proinflammatory capacity. In contrast, “tolerogenic” DCs would instead be regulatory, resulting in reduced or increased susceptibility to secondary infections (129).

For example, our team has shown that circulating DC from trauma patients express a tolerogenic transcription factor, Blimp1, characteristic of tolerogenic functions (130). In a mouse model of post-trauma immunosuppression, we demonstrated that administration of a TLR agonist could restore cytokine production from DC and improve the pulmonary response to pneumonia (131). This state of reversible DC paralysis is probably a consequence of an immunosuppressive microenvironment induced by TBI.

#### Natural killer cells

After severe TBI, the number of NK cells decreases rapidly (132). This decay persists for weeks (133) and correlates with trauma severity. After TBI, Mrakovcic et al. showed that the proportion of cytotoxic CD56^Dim^ NK cells decreases significantly at the expense of immunosuppressive CD56^Bight^ cells. On day four after TBI, the percentage of perforin-positive NK cells is also reduced compared to healthy controls (134).

A recent study looking at total NK cells within five days of trauma observed a transient decrease in the expression of the proinflammatory transcription factor T-bet and IFN-γ (135). Hence, IFN-γ secretion by NK cells is impaired after stimulation by S. aureus (136). Decreased IL-12R expression is also observed, associated with decreased STAT4 activation and IFN-γ synthesis. These TBI NK cells also show a deficiency in the activation marker CD25 and CD69 in the direct aftermath of trauma (136). There is an alteration in the expression of inhibitory and activating receptors KIR and NKG2D, and a hyporesponsiveness of TBI NK cells associated with spontaneous lysis, leading to a weak IFN-γ response and reduced degranulation in response to HLA-deficient target cells (122). IL-12 significantly triggered the IFN-γ and degranulation of TBI NK cells against HLA deficient cells, spontaneously (via inhibitory receptors) and via the antibody-dependent cytotoxicity pathway. Finally, IL-12 seems to be a new potential treatment available to overcome NK cell alterations in TBI patients (122).

#### CD4^+^ T cells

When activated after TBI, CD4^+^ T cells will differentiate into distinct T-helper (Th) cells. These subsets include Th1, Th2, Th17 and regulatory T (Treg) cells. Each of these subtype’s functions differently in the aftermath of TBI.

##### Th1/Th2

Under conditions of immune homeostasis, Th0 lymphocytes differentiate into Th1 and Th2 cells. Th1 and Th2 subtypes coexist in the aftermath of an inflammatory event and produce different groups of cytokines (137). CD4^+^ Th1 cells require IL- 12 and transcription factor T- bet in order to produce IL- 2, IFN-γ and TNF-α (138) and maintain the inflammatory functions of macrophages. CD4 +. Th1 cells permeabilize the BBB to secrete chemokines essential for leukocyte trafficking into the cerebral spinal fluid (139). Th2 cells require IL- 4 and the transcription factor GATA3 and produce neuroprotective cytokines, such as IL- 4, IL- 5, IL- 10, and IL- 13 and induce macrophages with rather anti-inflammatory phenotypes (140). After TBI, Th polarization is mediated by the TLR4 receptor on myeloid cells. Microglia secrete the pro-inflammatory chemokine CXCL10 which stimulates the infiltration of Th1 CXCR3 + cells (141). At the same time IL33 stimulates the production of Th2 cytokines necessary for wound healing in the case of CNS injury (142).

In the 24 hours following a TBI, there is a significant decrease in the number of circulating T cells (132) which affects both CD4 + T helper cells and CD8 + cytotoxic T cells. Several hypotheses for this lymphopenia is an accelerated apoptosis, but some authors have also suggested, based on experiments in mice, that high concentrations of catecholamines inhibit the exit of lymphocytes from lymph nodes (143). TBI is also associated with a loss of thymus mass which is associated overall with immunosuppressive features (118).

Following TBI, circulating T helper balance rapidly shifts toward Th2 (144, 145), increasing the susceptibility to infection (146). Several mechanisms participate in this phenomenon: first, TBI induces changes in the metabolism of the IL-2 cytokine. IL-2 is a potent Th1 cell growth factor essential in the cellular immune response (147). Early studies in polytrauma patients demonstrated a significant reduction in serum IL-2 and its soluble receptor in the weeks following TBI, suggesting immunosuppression of IL-2 regulated responses during this period (148). In addition to IL-2, altered productions of the proinflammatory cytokines IFN-γ and IL-12 are involved in post-traumatic immunosuppressive mechanisms. IL-12 is a promoter of IFN-γ secretion and NK cells cytotoxic activity, and decreased IL-12 secretion in the aftermath of TBI is a susceptibility factor for nosocomial infections (122). Schwulst et al. showed, for example, that IL-12 expression was decreased in TBI patients for up to 2 weeks after trauma (118). This significant alteration of the IL-12/INF-γ loop is one of the central mechanisms associated with inflammation-induced immunosuppression.

T cells from TBI patients also have higher expression of the PD1 receptor and the tolerogenic transcription factor BLIMP1 (149) making them less effective against hospital-acquired infections. This immunosuppressive microenvironment is found primarily in the lung parenchyma, where the PD1 ligand induces IL-10 overproduction and T cell apoptosis (150). And may partly explain the high incidence of PAVM in TBI patients. This phenomenon of “exhaustion” and the resulting lymphopenia is a risk factor for mortality in the ICU (151). This lymphopenia persists beyond six months in most patients and can be a source of chronic immunological disorders (152).

##### Treg

T reg are CD4 + T cells that express Foxp3 and CD25 (153) and are capable of differentiating into natural Treg cells (nTreg) and inducible Treg cells (iTreg); nTreg cells are the primary cells to infiltrate the CNS parenchyma after trauma (140). After reaching the site of injury, Treg secrete anti-inflammatory cytokines such as IL-10 and TGF-β, but also inhibit various immune cells such as circulating monocyte-derived macrophages or dendritic cells and limit neuroinflammation and brain damages. Treg also suppress other T helper cells by limiting the transformation of Th0 to Th1 and their brain infiltration (154). Consistently, the absence of Treg has been shown to correlate with increased brain damages and impaired functional outcome in C57BL/6 mice undergoing stroke (155) and increased T- cell infiltration and astrocyte proliferation in acute experimental TBI (154). After TBI, the level of circulating Treg is increased on day 1 and peaked on day 14. This increase in circulating Treg cells is correlate with functional outcome and may predict prognosis after TBI (156). However, an overwhelmed Treg response could lead to a state of immunosuppression and could increase susceptibility to infections as our team has demonstrated in sepsis (157).

##### Th17

Th17 lymphocytes are a subtype of CD4^+^ T cells depending on IL6 and TGFb as well as the transcription factor RORγT to develop (158). Animal models have shown that the increase in Th17 cells shortly preceded the increase in cytotoxic CD8 + T cells several days after TBI suggesting that Th17 cells may be responsible for cytotoxicity and neuroinflammation (159). Th17 cells promote cell migration across the BBB in some neuroinflammatory disease (160). and in a mouse model of TBI the Th1/Th17 polarization is a component of the intracerebral inflammatory response (161). In addition to sharing a similar developmental pathway, Th17 can differentiate into Treg, and Treg can determine Th17 orientation (162). High ratio of Th17 cells to Tregs are associated with post-traumatic infections (137), suggesting that tuning the Th17/Treg balance could be an important avenue of research to limit secondary damage induced by TBI. Supporting this hypothesis, the level of circulating Treg cells has been positively correlated with the neurological recovery of patients with TBI (156).

#### B cells

B cells are lymphoid cells that regulate the immune system both through direct interactions with the target and through the secretion of antibodies (163). These cells are poorly studied in TBI although B cells have been studied in many neuroinflammatory diseases and seem to show a specificity for the subacute phase of TBI. Our team was interested in the phenotype of B cells in TBI patients (164) and we showed that TBI patients have a significantly higher frequency of B cells with an activated profile at day 7 after injury. Our results also suggest that IL-10^+^ B cells may play a role in immunosuppression after TBI. Another study shows that B cells are activated by CNS antigens after TBI (165). Subsequently, the study of B cells in mouse models showed that B cell-deficient mice exhibit an enhanced immune response after TBI (159), indicating a potential protective role for B cells in TBI. The importance of immunosuppressive regulatory B cells and their role in maintaining the regulatory T cell compartment is becoming increasingly well documented (166). One hypothesis for the role of B cells in TBI is the secretion of anti-inflammatory cytokines (e.g., TGF-β and IL-10) that could limit microglia overactivation and decrease macrophage and Th cells infiltration. Secretion of anti-inflammatory mediators would promote parenchymal healing through the induction of a protective microenvironment as demonstrated in an animal model of autoimmune neuroinflammation, where B cells modulated neuroinflammation and limited Th1/17 responses via TGF-β production (167). In mouse models of stroke, intravenous infusion of IL-10-producing B cells was observed to reduce neuroinflammation and infarct volume (168). In a mouse model of TBI, intraparenchymal injection of mature B cells improves structural and functional outcome, and lesion volume in mice treated with B cells was significantly reduced by 40% at 35 days after TBI (169). T-cell activation, astrogliosis, and microglial activation were also reduced. Lymphocytes appear to have an interesting role in TBI and may serve as a candidate for future study in the subacute phase.

## Diagnosis and therapeutic approaches

Immune changes after TBI are pleiotropic: In the direct aftermath of TBI, brain inflammation leads to neuron and white matter damages. After major activation of immune cells, the CARS leads to tolerogenic state which induces major sensitivity to secondary nosocomial infections. However, immune cells activation can last many years after TBI (110) and contribute to chronic neuroinflammation process. This persisting TBI-induced neuroinflammation is associated with poor outcomes (170) and neurodegenerative post traumatic disorders (171).

Therefore, there are multiple approaches to modulate immune cells in TBI. In the early phase, it aims to reduce the cerebral and systemic inflammatory reaction. Subsequently, the challenge is to restore peripheral immune functions in order to avoid secondary infections and organ failures in ICU. Finally, in the late phase, therapeutics can be developed to limit chronic cerebral inflammation and chronic neurologic disorders.

The challenge for ICU physicians caring for TBI patients would be to detect the stage of the disease via the implementation of biomarkers or imaging and thus be able to apply personalized medicine to improve outcomes.

### Diagnosis of neuroinflammation and immune disorders

#### Biomakers of immune dysfunction

The occurrence of infectious complications in TBI patients remains frequent without us being able to predict or prevent their occurrence. The development of the “personalized medicine,” particularly in oncology, made possible to adapt chemotherapy to the tumor and to the genetic characteristics of the patients, thus avoiding the principle of “one size fits all” to a tailored approach (172). In TBI patients, research in this area is in development (173). In ICU patients, the challenge remains to find biomarkers of occurrence of nosocomial infections in order to develop adapted immunomodulatory treatments. Like post-traumatic immunosuppression, sepsis-induced immunosuppression is the subject of research to establish biomarkers of this immunosuppressive state (174). Decreased monocyte HLA-DR membrane expression was one of the first markers used to define post-sepsis immunosuppression (175) and its persistence has been highlighted as a risk factor for mortality (176). Others biomarkers have subsequently been highlighted such as the decrease in TNF-α production by leukocytes after LPS stimulation (177) or the increased PD-L1 membrane expression by circulating monocytes inducing a state of CD4 T cell tolerance (178). High throughput cell sorting devices have provided insight into heterogeneity of the host response to sepsis or trauma. From now, biomarkers allow classification of patients into different phenotypes and thus facilitate the identification of distinct subgroups (or endotypes) and secondary tailoring of immunomodulatory therapy (179). Thus, studies have identified genetic variants that may contribute to an impaired immune status observed in septic patients with an unfavorable course (180). However, immune dysfunctions in ICU patients is not limited to patients with sepsis, but also occurs in patients with sterile inflammation where the usefulness of immune dysfunction markers of neutrophils, T cells and monocytes may also allow prediction of ICU complications (181). In trauma patients, the wide variety of traumatic injuries and injured organs induces a wide range of inflammatory and anti-inflammatory responses. Precision medicine seems very promising for these patients. New technologies such as mass cytometry promise an analysis of many cellular markers and will allow the phenotyping of immune response to trauma (135). Publications focusing on the use of transcriptomic data on PBMC samples from trauma patients to highlight genomic signatures that correlate with poor patients outcome (182) or the occurrence of post-trauma nosocomial infections (183). Similarly a study conducted in burn patients, developed a blood transcriptomic panel of biomarkers to predict the occurrence of infections (184). Another recent study using genome-wide information analyses identified several leukocyte signatures associated with susceptibility to infection (185). However, the impossibility of the routine use of these technics as well as their cost does not allow the clinical use of the resulting biomarkers. Our team published in 2019 (186) a work aiming to develop an easy-to-use biomarker to predict the occurrence of VAP in TBI patients. This biomarker is a combination of two biological parameters (CRP and total cortisol) which reflect the disorders between pro- and anti-inflammatory mechanisms. In our study, this biomarker discriminated patients who could benefit from treatment with steroids to prevent the occurrence of VAP. Others easy-to-use biomarkers have been evaluated in TBI patients. As with patients in septic shock, decreased membrane expression of HLA DR on leukocytes is also associated with the occurrence of sepsis after severe trauma (187). Hildebrand et al. showed a correlation between increased serum IL-8 levels and the development of ARDS in polytrauma patients (188). In other work, elevated IL-6 levels in serum of polytrauma patients were associated with the occurrence of multiorgan failure (189). Cohen et al. demonstrated that HMGB1 is released early after severe trauma and correlates with organ failure, and with the occurrence of pulmonary infection (190).

Biomarkers are essential diagnostic and prognostic tools in TBI patients because the intensity of the initial inflammatory state has consequences on the subsequent complications. Biomarkers research has produced a multitude of molecular and genetic signatures but the exact purpose and therapeutic consequences of using these diagnostic tools have yet to be defined (191).

#### Imaging diagnosis

The use of non-invasive imaging devices to diagnose neuroinflammation is a promising area of research. These tools are not useful in the early phase of TBI because of the direct traumatic lesions (blood, CSF.) making their use impossible. From 2 weeks after the TBI, molecular imaging of microglia and brain macrophages like positron emission tomography (PET) and magnetic resonance (MR) imaging can be interesting. PET using radioligands specific of microglia activation has shown good sensibility and specificity regarding cerebral inflammation evaluation (192). Advances in magnetic resonance imaging of microglia using iron oxide nanoparticles and ultra-small super paramagnetic particles that are phagocytosed are also in development. The single photon emission computed tomography tracer 123 I-CLINDE, which visualizes translocator protein (TSPO), a protein upregulated in active immune cells has for example allowed to diagnose neuro-inflammatory states persisting 2 weeks after the trauma and a correlation with a poor neurological outcome (193). The combination of PET and MR allows the simultaneous quantification of the volume, localization and intensity of microglia inflammation and thus the mapping of the inflammatory damage. In the future, the combination of blood biomarkers based on the host response to trauma with advanced imaging techniques will allow the development of treatments adapted to the neuroinflammatory and immune status of patients.

### Therapeutic approaches

To correct posttraumatic immunosuppression and prevent nosocomial infections or to limit chronic neuroinflammation, many therapies have been evaluated in recent years. They aimed either to limit initial and chronic inflammation, including the use of low-dose glucocorticoids (4, 194), or to restore antigen-presenting functions or cytokine secretory capacities through the use of IFN- γ, GM-CSF (195), or interleukin-12 (130) (Table 1).

**TABLE 1:** Potential immunomodulating agents for the treatment of neuroinflammation and post-traumatic immunosuppression.

Limitation of initial inflammation	Agent	Potential therapeutic impact	References
	Steroids	↓Inflammatory cytokines ↓Adhesion and recruitment of T cells and neutrophils ↓HPA stimulation by IL-6 ↓DAMPs & PRRs	(4, 127, 186, 197)
	IL-1R inhibitor	Inhibition of MAPK and NF-κB pathway ↓Microglial activation ↓Inflammatory cytokines	(202–206)
	Anti-TNF- α	↓TNF-α-induced activation of glia ↓Brain inflammation ↓BBB breakdown	(208–210)
	Neurokinine 1 antagonist receptor	Blockage of substance P binding ↓BBB breakdown ↓Vasogenic oedema	(212–214)
	Coagulation pathways (Tranexamic acid, aprotinin, antithrombin 3)	Clot stabilization ↓Inflammatory effects of plasmin Inhibition of thrombin	(215–218)

**Restoration of innate immune cells functions**	**Agent**	**Potential therapeutic impact**	**References**

	Steroids	↑Phagocytic capacities of neutrophils ↑INF-γ and IL-12 ↓IL-10 ↑T cells recruitment	(198, 199, 201)
	Interferon γ	↑Others inflammatory cytokines ↑HLA-DR expression Restores macrophages, monocytes & neutrophils inflammatory activities	(219–228)
	IL-12	Restores the loop between DCs and effectors cells Restores Inf γ production Restores NK cells cytotoxicity	(122, 126)
	GMCSF/ GCSF	↑Myelopoiesis/↑phagocytosis ↑Cytokines and adhesion molecules ↑HLA-DR expression on antigen-presenting cells	(229–234)

**Limitation of chronic neuroinflammation**	**Agent**	**Potential therapeutic impact**	**References**

	Neural stem progenitor cells	Secretion of glial cell-derived neurotrophic factor Differentiation into neurons Secretion of neuroprotective factors	(249, 250)
	Mesenchymal stem cells	Secretion of neurotrophic factors ↓Angiogenesis via VEGF ↑Neuroinflammation	(252–254)

#### Limitation of initial inflammation

##### Corticosteroids

In TBI patients, early administration of corticosteroids seems to be an attractive therapeutic approach. The administration of corticosteroids in this context of relative post-traumatic immunosuppression may be a paradox, but the effects of corticosteroids, especially at low doses, are pleiotropic. Indeed, in TBI patients, corticosteroids may decrease immunosuppression and the occurrence of nosocomial infections by two mechanisms:

• During the inflammatory phase, steroids decrease the secretion of proinflammatory cytokines, the expression of PRRs, and the adhesion and recruitment of T cells and neutrophils (196). The introduction of corticosteroid therapy may also reduce the stimulation of the IL-6-dependent HPA axis, limit the anti-inflammatory response, and thus limit the prevalence of VAP in patients with exacerbated inflammation (197). In a recent ancillary study to the Corti TC trial (4), we showed that hydrocortisone decreases the rates of hospital-acquired pneumonia in patients with a significant imbalance between pro- and anti-inflammatory mechanisms (186). TBI induced-immunosuppression is partly due to NK cell damage to dendritic cells via an IL-10-dependent mechanism, and glucocorticoids limit this immunosuppressive loop (198). We thus propose that the anti-inflammatory effects of early injection of steroids prevent the development of the CARS, thus hasting the return to immune homeostasis and decreasing the susceptibility to secondary infections.
• Glucocorticoids modulate dendritic cells during and after inflammation to restore their central role in the immune response (127), thereby reducing trauma-induced tissue damage and susceptibility to bacterial infections. Low-dose glucocorticoids also act post-trauma by restoring the effector capacities of innate immune cells affected by TBI. Shortly after the inflammatory phase, the administration of corticosteroids decreases anti-inflammatory cytokines (IL-10) (198), upregulates PRRs & cytokine receptors, increases phagocytic capacity (199) and increases leukocyte recruitment (200). Low-dose glucocorticoids also enhance the phagocytic abilities of neutrophils and increase interferon- γ and interleukin-12 production, which are one of the main proinflammatory cytokines involved in host defense against infection (201).

All these arguments suggest that in patients with severe TBI and an exacerbated overcompensated inflammatory response, glucocorticoids may be beneficial in preventing nosocomial infections. The challenge remains to find biomarkers to identify these highest-risk patients who may benefit from this corticosteroid therapy.

##### IL-1R inhibitor

IL1 β is a pleiotropic cytokine secreted immediately after TBI. In the brain, IL1 β is secreted by microglia and induces the inflammatory cascade by stimulation of astrocytes and other brain cells. IL-1β can activate both the MAPK and NF-κB pathways. Activation of these pathways results in the transcription of proinflammatory genes and amplification of the cerebral inflammatory response. Recombinant interleukin-1 receptor antagonist (IL1ra) is an attractive therapeutic option to reduce early brain inflammation and limit secondary damages. Experimental studies demonstrated that blockade of IL-1β through treatment with IL-1Ra results in inhibition of inflammatory cascades, microglial activation, and proinflammatory cytokine expression (202). In mice, Anakinra (IL1Ra) improved performance on cognitive tasks (203) but other experimentations could not confirm these findings (204). IL1ra use has demonstrated benefits in chronic cerebral pathologies by inhibiting the IL1 receptor-mediated inflammatory cascade (205). In human TBI, it was shown to be safe and modify the acute neuroinflammatory response in a phase II single-center RCT (206).

##### Anti-TNF-α

TNF-α is a ubiquitarian proinflammatory cytokine localized in the plasma membrane as transmembrane TNF-α and is highly expressed in brain cells (207). TNF activates two receptors: TNFR1 and TNFR2 (208). TNF-α is involved in different aspects of TBI pathophysiology. It activates multiple inflammatory pathways (NF-kB, MAPK) to intensify inflammation and can induce the breakdown of the blood-brain. The use of Anti-TNF α in TBI patients is justified by the early or late reduction of cerebral and then systemic inflammation, in part due to blocking TNF-α-induced activation of glia (209). Two studies reported improved cognitive and motor outcomes in TBI patients having received single-dose peri-spinal administrations of etanercept (210). Still, these studies were conducted at a distance from the TBI.

##### Neurokinine 1 antagonist receptors

TBI induces immediate vasogenic edema which requires an increased BBB permeability to serum proteins and immune cells. Increased BBB permeability after TBI with subsequent edema formation has been recently linked to substance P (SP) release (211). Neurokinin 1 receptor antagonist (NK1) acts by blocking the binding of SP to neurokinin 1 receptors. In rats models, inhibition of SP action by administration of the NK1 antagonist at 30 mins after trauma attenuated vascular permeability, edema formation and also improved both motor and cognitive neurologic outcomes (212). Administration of NK1 antagonist reduced brain edema and intracranial pressure in other murine models (213).

Finally, authors hypothesize that the increase in SP-mediated protein transcytosis increases vascular permeability, contributing significantly to the development of increased intracranial pressure and a major influx of circulating immune cells via the BBB. Administration of NK1 antagonists reduces this protein transcytosis, decreasing vasogenic edema and reducing BBB permeability (214).

##### Coagulation pathways

The coagulation and immune systems interact following the TB mainly through the activation of complements. Tranexamic acid competitively inhibits the conversion of plasminogen to plasmin, thereby inhibiting fibrinolysis. Its efficacy was demonstrated in the CRASH 2 trial (215) in polytrauma patients. While the better survival in the tranexamic acid group was partly due to decreased bleeding and faster resolution of hemorrhagic shock, a military observational study (216), showed that its use also limited the inflammatory response caused by fibrin degradation products. The use of other factors affecting coagulation, such as aprotinin (217) or antithrombin-3 (218) for anti-inflammatory purposes, such as decreased IL-8 production, did not yield the expected results.

#### Restoration of innate immune cells functions

##### Interferon γ

As seen previously, TBI induces an inability of the antigen-presenting cells to present antigen to the effector T cells, notably via the human leukocyte antigen DR (HLA DR). Numerous studies, both in the laboratory and in the clinical setting, have focused on HLA-DR, as its expression on monocytes has been shown to reflect the host’s ability to present antigens (219, 220). Polytrauma patients with impaired antigen-presenting capacity develop more infections than patients with normal HLA-DR levels (221). In parallel, interferon-γ upgrades the antigen-presenting capacity of monocytes via HLA DR in mice (222) and in humans (223). Recombinant IFN-γ has been evaluated in 4 studies in trauma patients. Daily administration of IFN-γ at a dose of 100 μg subcutaneously had a positive effect on mortality (224) and inhaled IFN-γ prevented the occurrence of infection (225). However, other trials did not show a beneficial effect (226), as restoring HLA DR expression was not necessarily correlated with restoring immune functions altered by the trauma (227). Recently this therapy has been evaluated in post-septic immunodepression, demonstrating promising results when administrated lately (228).

##### GM-CSF and immunoglobulins

Acquired neutrophil dysfunction is central to immune system failures after TBI. Among them, impairment of neutrophil phagocytosis is associated with occurrence of nosocomial infections. (92) and results in an alteration of the C5a-CD88 function (229). Granulocyte-macrophage colony-stimulating factor (GM-CSF) is a multifunctional cytokine that regulates inflammatory responses and is involved in a wide range of biological processes in both innate and adaptive immunity in response to danger signals (230). In critically ill patients with sepsis-induced immunosuppression, GM-CSF restores monocyte expression of HLA-DR (231) and improves neutrophils phagocytic capacity in patients with impaired neutrophil phagocytosis (232). GM-CSF have been studied on cells from polytraumatized patients showing an effect on the reactivity of innate immunity cells (233). However, these effects have never been found *in vivo* in trauma. GC-SF which stimulates stem cells to produce granulocytes was tested in 61 adults with GCS score ≤ 8, showed a dose-dependent increase in neutrophil count and a significant decrease in bacteremia incidence but no effect on strong ICU outcomes (234).

#### Immune modulation via metabolic pathways

Recent advances in cellular metabolism allow us to consider new therapies for inflammatory diseases. Immunometabolism is the study of immune cell metabolism and aim to develop therapies which modulate metabolism and regulate endogenous metabolites with anti-inflammatory effects (235). By modulating various metabolic pathways of proinflammatory immune cells the aiming is to promote a more anti-inflammatory phenotype notably by Treg cells and anti-inflammatory macrophages development, whilst suppressing differentiation of Th17 cells and the more inflammatory M1 macrophages. In sepsis, immunometabolism is more advanced than in trauma patients (236). In sepsis, CYT107 has an impact on immunometabolism *via* improving mTOR signaling, GLUT1 surface expression, and glucose uptake by T cells (237) and targeting this metabolism pathway showed promising results in a phase II clinical on patients in septic shock (238). In TBI patients, several therapeutic modulating metabolic pathways have shown an interesting effect, most of them on animal models. Dimethyl fumarate (DMF) has both immunomodulatory and antioxidant properties by activation of the Nuclear factor erythroid 2-related factor 2 (Nrf2) in different cell types which triggers antioxidant gene expression. DMF treatment after TBI in mice prevents depletion of antioxidative brain glutathione, reduces brain tissue loss and confers neuroprotection (239). Metformin is a treatment prescribed worldwide for its role in glucose metabolism in type 2 diabetes patients. In mice TBI model, metformin treatment increases ramified microglial morphology with longer total branch lengths indicating reduced neuroinflammation (240). Metformin also reduced cytokine production in a rat model of TBI by suppression of NF-κB and MAPK activation (241). Rapamycin targets the PI3K/Akt pathway which is a central metabolic pathway in immune cells inflammation (242). The target of rapamycin, commonly known as mTOR regulates translation and cell division. In TBI model, Rapamycin inhibits p70S6K phosphorylation, reduces microglia/macrophages activation and increases the number of surviving neurons at the site of injury (243). Treatment with mTOR inhibitors ameliorate the post TBI neuroinflammation, limit neuronal apoptosis and astrogliosis (244). There are many other metabolic pathways that could be targeted to limit the inflammatory and dysmetabolic consequences induced by TBI (245).

#### Limitation of chronic neuroinflammation

In recent years, new therapeutic approaches have emerged to limit the chronicity of neuroinflammation linking to chronic neurodegenerative disease such as Parkinson’s or Alzheimer’s (246). Limiting neuroinflammation is therefore a major issue in the management of these patients and an important field of research. The study of pluripotent stem cells is an avenue of research in many inflammatory diseases such as neurodegenerative disease, diabetes or auto-immune disease (247). In TBI patients, these therapeutics could be very effective (248). There are 2 main stem cell types (1), the neural stem progenitor cells (NSPCs), embryonic stem cells derived from fetal tissue (extremely difficult to obtain in practice) and (2) mesenchymal stem cells (MSCs) which can be found in most tissues and are typically harvested from adipose tissue, peripheral blood, human umbilical cord blood (hUCB), or bone marrow (BMSCs). Research on NSPCs is limited due to the technical difficulty but is promising because of the ability to differentiate into neurons in the injured region, as well as through secretion of glial cell-derived neurotrophic factor and other neuroprotective factors (249). The use of human NSPCs in rats showed an incorporation at the site of inflammation and cells remain in the tissue 2 weeks after injection allowing improvement in the animal’s neurological recovery (250). No large-scale trials of NSPCs in TBI patients have yet been conducted. MSCs do not have the capacity to differentiate into neurons but have the ability to modulate the inflammatory response and to secrete neurotrophic factors which promote the protection and development of neurons (251). After intravenous injection these cells go to the injury site guided by the secretion of chemokines and cytokines by the activated immune cells. The effect of MSCs is anti-inflammatory; the use of autologous BMSCs in severe TBI patients demonstrated structural neurological preservation correlating with functional outcomes and a downregulation of key inflammatory cytokines (IL-1β, and IFN-γ) (252) BMSCs therapy for seven patients, using intracerebral transplantations and intravenous injections, showed improvements in neurologic recovery (253). The use of Non-Autologous MSCs, notably hUCB MSCs, is more easy and shows promising results (254). Further studies are needed to understand the mechanisms by which stem cell therapies promote recovery following TBI, as well as evaluate the effectiveness of these therapies in larger cohorts.

## Conclusion

TBI initiates both local and systemic inflammatory responses which aim at enabling brain-tissue healing while limiting the risk of overwhelming inflammation. Given the strong interplay between the brain and peripheric organs in health conditions, the demonstration that the time course of the inflammatory responses in the brain and systemic compartment are highly correlated was almost expected, but still suggests that immune interventions have the potential to enhance neurological outcomes after severe brain injury. While several approaches can be proposed for pilot evaluations in humans, it will be crucial to deeply characterize both the systemic and brain effects of such approaches to understand potential unexpected effects and define subgroups of responders and non-responders.

## Author contributions

MB and AR wrote the draft. KA extensively reviewed the manuscript. All authors approved the manuscript before the submission.
